# 3D Printed Fractal-like Structures with High Percentage of Drug for Zero-Order Colonic Release

**DOI:** 10.3390/pharmaceutics14112298

**Published:** 2022-10-26

**Authors:** Vicente Linares, Ángela Aguilar-de-Leyva, Marta Casas, Isidoro Caraballo

**Affiliations:** Department of Pharmacy and Pharmaceutical Technology, University of Seville, 41012 Seville, Spain

**Keywords:** 3D printing, FDM, IVF, extruded filaments, fractal analysis, colonic drug delivery, zero-order release

## Abstract

Colonic drug delivery of drugs is an area of great interest due to the need to treat high prevalence colonic local diseases as well as systemic conditions that may benefit from the advantages associated to this route of drug administration. In the last decade, the use of 3D printing technologies has expanded, offering the possibility of preparing personalized medicines in small batches directly at the point of care. The aim of this work is to design a high drug loaded 3D printed system prepared by a combination of Fused Deposition Modelling (FDM) and Injection Volume Filling (IVF) techniques intended for zero-order colonic drug release. For this purpose, different batches of binary and ternary filaments based on the thermoplastic polyurethane Tecoflex EG-72D (TPU), theophylline anhydrous (AT) as model drug, and magnesium stearate as lubricant have been developed and characterized. Filaments with the highest drug load and the best rheological properties were selected for the manufacture of a printed fractal-like structure based on multiple toroids. This design was proposed to provide high surface area, leading to increased drug release and water uptake in the colonic region. This structure was 3D printed by FDM and then coated in a unique step by IVF technology using the enteric polymer DrugCoat S 12.5. This way, an additional coating process is avoided, reducing costs and production time. Studies of drug release confirmed the ability of the structures to provide a five-hour period of constant drug delivery in the colonic region.

## 1. Introduction

The colonic region of the gastrointestinal tract constitutes an important target for therapeutic intervention due to the need to treat local colon diseases such as inflammatory bowel disease, including ulcerative colitis and Crohn’s disease, irritable bowel syndrome, local infections, and carcinoma [1]. Pathologies such as these can be treated locally, reducing the amount of drug necessary and avoiding systemic absorption, while also preventing possible adverse side effects [2]. Moreover, in the last years, there is an increasing interest in the colonic drug delivery intended to treat systemic conditions. The colonic area offers important advantages, such as prolonged transit time, low level of peptidases, and good response capacity to penetration enhancers [3]. 

There are several strategies to target colon release [1,4,5]. Nevertheless, most commercially available products are based on pH-dependent systems. These systems employ enteric polymers, such as polymethacrylates that dissolve above a concrete pH value and prevent the early drug release in the upper GI tract, allowing drug release in the distal small intestine and colon. Other formulation approaches employed to achieve colon targeting are time-controlled systems, enzymatic triggered release systems or even a combination of several approaches such as pH and enzymatic triggered release systems [6]. 

Nowadays there is a great emphasis on patient-centered dosing of medicines instead of mass production. The need for individualized medicines is due to differences in the patient’s age, weight, body surface, drug clearance capacity, and severity of the disease. 3D printing offers the possibility of preparing personalized medicines in small batches directly at the point of care, such as within hospital or community pharmacies. The dosage form could be designed to contain an appropriate dose, drug combinations, formulation type and/or different geometric forms or aesthetics that are custom-made to suit the patient’s needs [7,8]. 

Although there are an important number of 3D printing technologies that can be used to produce a wide range of different dosage forms, Fused Deposition Modelling (FDM) is one of the most frequently employed [9]. In fact, it is considered the lowest cost and easily accessible 3D printing technique [10]. In FDM printers, thermoplastic polymers are used [11] in form of filaments that are melted and extruded through a heated nozzle and settled on a heated platform layer by layer [12]. These layers solidify as soon as they are deposited on the platform, allowing the next layer to be deposited on top of it until the structure is completed [13]. This technology offers the possibility of modulating the dissolution profile of the drug by varying parameters such as the type of polymer used, the dimensions of the system, the infill density, the percentage of drug, or the geometry of the dosage form [14,15]. 

A delayed release pattern can be achieved using FDM 3D printing through different strategies [16,17,18,19]. Some of them are the design of a monolithic system employing enteric polymers like hydroxypropyl-methylcellulose acetate succinate (HPMC-AC) or making use of a dual FDM 3D printer to construct a shell-core structure based on Eudragit L100-55 filaments as an enteric surrounding shell [20]. Additionally, FDM can be coupled with a syringe-based dispenser to inject liquids or semisolid components at room temperature in the extruded scaffold. This way, a following step of coating of the 3D printed cores with an enteric polymer in a fluid bed coater is avoided. In previous studies, this complementary technique known as Injection Volume Filling (IVF) has been successfully employed to obtain colon drug delivery systems employing the pH sensitive acrylic polymer Eudragit FS30D. The systems prepared by the combination of FDM and IVF consisted of FDM extruded PLA scaffolds in which a very low drug dose and the enteric polymer are injected by means of IVF [19,21]. 

In this study, we propose the use of the combination of IVF and FDM in order to develop a colonic dosage form with a higher concentration of API. For this purpose, it is necessary to prepare high drug loaded filaments that can be obtained making use of a lubricant. The use of lubricants has demonstrated to improve the extrusion and printability of filaments, allowing a better rheology and facilitating the passage through the nozzle of the printer when high drug content is used [15,22,23]. 

Our research group has experience in the development and evaluation of filaments, especially in the application of fractal analysis to predict the printability of the filaments. It has been demonstrated that this tool is a non-destructive, non-expensive, and fast instrument to determine the printability [15,24].

The aim of this work is to develop a simple method for obtaining 3D printed systems with high drug load and zero-order colonic drug release. Filaments based on the thermoplastic polyurethane polymer EG72 intended for prolonged drug release and anhydrous theophyllineas model drug will be prepared and characterized. FDM 3D printed fractal-like structures based on multiple toroids will be proposed in order to provide high surface area -and therefore increased drug release-, and to favor the water uptake in the colonic region thanks to the pores. This structure will be coated by IVF technology using an enteric liquid polymer, which dissolves above pH 7. Studies of the drug release will be carried out with the aim to evaluate the ability of the structures to provide colonic zero-order drug release.

## 2. Materials and Methods

### 2.1. Materials

Anhydrous theophylline (AT) (batch 151209-P-1, Acofarma, Barcelona, Spain) was used as a model drug. The medical grade TecoflexTM EG-72D thermoplastic polyurethane (TPU) supplied by Lubrizol Advanced Materials (Barcelona, Spain) was used as a matrix-forming polymer. Magnesium stearate (batch 201081, Acofarma, Barcelona, Spain) was used as lubricant. DrugCoat S 12.5 was kindly supplied by Vikram Thermo (Ahmedabad, India) and was employed as solubility pH-dependent polymer for colonic release.

### 2.2. Methods

#### 2.2.1. Preparation of the Physical Mixture

TPU pellets were frozen using liquid nitrogen and pulverized in a mill (Retsch ZM 200, Haan, Germany) using a 1.0 mm output sieve (mean diameter 227.9 ± 166.0 μm). An AT particle size less than 180 μm (mean diameter 121.5 ± 45.0 μm) was used. Binary mixtures of AT and polymer powder and ternary mixtures adding magnesium stearate were mixed for 15 min in a Turbula mixer (Willy A. Bachofen, Basel, Switzerland). Table 1 shows the composition of the different lots prepared. The optimum blend time was selected according to a previously published study [25]. 

#### 2.2.2. Extrusion Process

The physical mixture was extruded using a single screw Noztek Pro extruder (Noztek, Sussex, UK) adapting the method previously described by Linares et al. [24] at an extrusion temperature of 130 °C for the binary mixtures. For ternary mixtures, the extrusion temperature decreased as the AT concentration increased. In this way, batch T20 was extruded at 150 °C, batch T30 was extruded at 145 °C, and batches T40–T60 were extruded at 135 °C. The screw speed for all batches was 33 rpm. The diameter of the nozzle was 1.75 mm. The extruder was preheated for 30 min to establish thermal equilibrium before extrusion. The diameter of the obtained filaments was measured using a digital micrometer (Comecta, SA, Barcelona, Spain) in order to verify that the required value of 1.75 mm of the feedstock material for 3D printing was achieved. After that, the filaments were stored in an appropriate packaging at 25 °C before the printing process.

#### 2.2.3. Mechanical Property Testing of Filaments

The mechanical properties of filaments were evaluated using a Texture Analyser TA.XTPlus (Stable Micro Systems, Godalming, UK) at room temperature. A stiffness test was applied to measure the toughness of the filaments following the method described by Xu et al. [26]. The test was carried out in triplicate for each filament. 

Filament samples were cut into 6 cm pieces and placed on top of the Texture Analyzer platform. The Texture Analyzer was equipped with a Warner Bratzler blade. Trigger force was set to 50 g, and the blade was set to cut the filament until 1 mm in distance (57% strain) at a speed of 2 mm/s. The maximum force and fracture distance were recorded using the Texture Analyzer software ‘Texture Exponent’ version 6, 1, 20, 0. The area under curve (AUC) and maximum stress were calculated using the Macro program in Texture Analyzer software. Three anchors are inserted in the plot using the Macro program in the texture analysis software. Anchor 1 is set at the starting point of strain, anchor 2 is at the max strain, and anchor 3 is at the end of the test when the stress approached zero. The AUC between every two anchors is recorded. The sum of AUC between anchor 1 & 2 and anchor 2 & 3 is named ‘toughness’ and is expressed in kg/mm^2^.

#### 2.2.4. Fractal Analysis

The box-counting technique is the methodology by which fractal analysis is performed and is based on the analysis of the perimeter from binary images, considering every pixel as a ‘box’. The fractal analysis was previously carried out by Linares et al. [24]. Briefly, with this technique we can calculate the fractal dimension value following Equation (1):

(1) $$N\left(\lambda \right)={C\lambda }^{-D}$$

where *N* is the number of boxes, C is a constant of proportionality, λ is the length of the box side, and *D* is the fractal dimension.

The roughness of the filaments was studied by taking photographs with a stereo microscope SMZ800N (Nikon Instruments Inc., Melville, NY, USA). Sixteen images were studied for each batch prepared [24].

#### 2.2.5. 3D Printing Process

3D printed systems were manufactured with T50 filaments that contain the largest amount of AT that can be printed. These filaments contained AT, thermoplastic polymer and magnesium stearate (50-45-5% *w*/*w*). Two 3D printing technologies have been combined: FDM technology was used to print the fractal-like structure on a Raise3D Pro2 printer (Raise3D Technologies, Inc., Irvine, CA, USA). The final drug delivery system was obtained thanks to the technology IVF carried out in a bioprinter (Regemat 3D, Granada, Spain) which deposits the enteric coating polymer (DrugCoat S 12.5) over the previously printed structure. This coating polymer was already charged into the syringe of the bioprinter. The software BlocksCAD (BlocksCAD Inc., Burlington, MA, USA) was used to design the 3D printed systems. 

**Figure 1:**
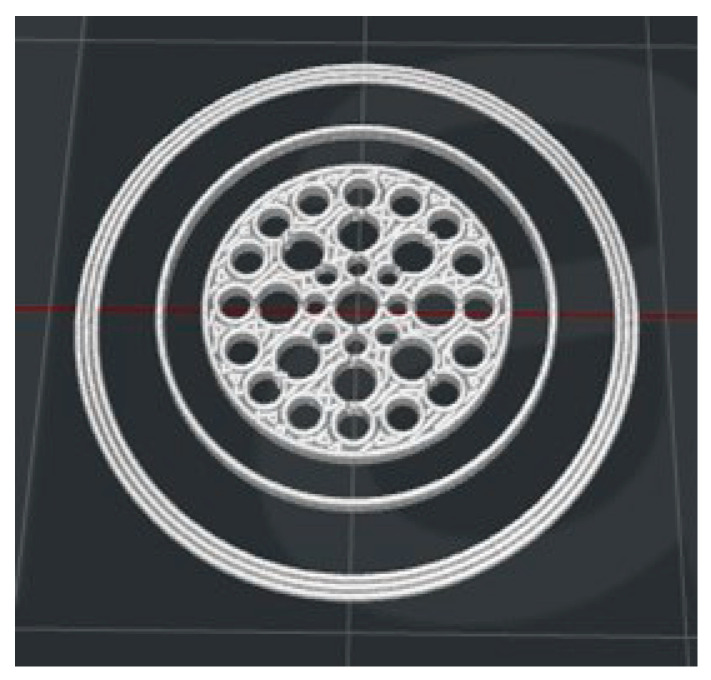
Digital design of the Fractal-like FDM structure.

The printing settings for the Raise3D Pro2 printer (Raise3D Technologies Inc., Irvine, CA, USA) were as follows: nozzle diameter of 0.4 mm, layer thickness 0.05 mm, nozzle temperature 220 °C, heated bed temperature 55 °C and printing speed 60 mm/s. The 3D printed structures consisted of 32 radial pores surrounding a central pore with 1 mm of height and 15 mm of diameter (see Figure 1).

The printing settings for the IVF process were: tip diameter of 0.58 mm, deposit speed 1 μL/s, retract speed 1 μL/s and purge 50 μL. The final amount of polymer deposited was 500 μL. 

Once the structures were completely printed, they were dried at room temperature for 24 h to generate the enteric coating layer.

#### 2.2.6. Physical Tests of Printed Systems

Six 3D printed structures were weighted in a precision balance Scaltec type SBC 31 (Gram Precision S.L., Barcelona, Spain) and the thickness and diameter of six 3D printed structures were measured using a digital micrometer (Comecta, SA, Barcelona, Spain).

#### 2.2.7. Thermal Analysis of Filaments

##### Differential Scanning Calorimetry

Differential Scanning Calorimetry (DSC) was used to study the thermal behaviour of the pure components, physical mixture (ternary mixtures in the adequate proportion to prepare T50 filaments), T50 filaments and 3D printed systems in order to confirm the stability and compatibility of the AT and the excipients. A DSC Q20 V24.11 Build 124 (TA Instruments, New Castle, DE, USA) was used. Approximately 8–12 mg of each sample were placed in crimped hermetical aluminum pans. All the samples were heated at 30 °C under a dry nitrogen flow (100 mL/min). A ramp rate of 10 °C/min was used until the end temperature of 350 °C was reached. Data were analyzed at the Functional Characterization Service of the Center for Research, Technology, and Innovation of the University of Seville (CITIUS). A TA Instruments Universal Analysis software was used. Thermograms were analyzed to detect thermal events.

##### Thermogravimetric Analysis 

Thermogravimetric analysis (TGA) was performed by a thermal analyzer (SDT Q600 V20.9 Build 20, TA Instruments, New Castle, DE, USA) to analyze the thermal decomposition of the pure components, physical mixture (ternary mixtures in the adequate proportion to prepare T50 filaments), T50 filaments, and 3D printed systems. Samples (about 4–11 mg) were weighed accurately and placed in an aluminum crucible. Samples were heated from room temperature to 800 °C at a heating rate of 10 °C/min and a nitrogen gas purge of 100 mL/min. The data was analyzed at the Functional Characterization Service of the (CITIUS by TA Instruments Universal Analysis software.

#### 2.2.8. Scanning Electron Microscopy (SEM)

The surface of T50 filaments, the FDM printed structures as well as the transversal section of the coated structures were evaluated using a FESEM (Field Emission Scanning electron microscope) Schottky type (Thermofisher, Eindhoven, Holland) operating at 5 kV in the Microscopy Service of the CITIUS in the Universidad de Sevilla. Samples were previously coated with a 10 nm-thin Pt layer with a Leica ACE 600 high vacuum sputter coater.

#### 2.2.9. X-ray Tomography

In order to analyse the homogeneous distribution of the drug in the 3D printed system, X-ray tomography was performed by the X-ray Laboratory Service of the CITIUS in the Universidad de Sevilla using a Zeiss Xradia 610 Versa (Zeiss, Oberkochen, Germany). The scan was conducted at a voltage of 30–40 kV. 3D printed structures were scanned using no filter, an optical magnification of 4× and 0.4×, and a pixel size of 0.8869 µm and 0.3911 µm, respectively. The exposure time was 15 s for images at a magnification of 4× and 5 s for images at a magnification of 0.4×. Image reconstruction was performed using Reconstructor Scout-and-Scan v.16.0, 11.592 software and exported as a 16-bit tiff file for visualization.

#### 2.2.10. Dissolution Testing of Filaments

Dissolution studies of B50 and T50 filaments were carried out in a USP Apparatus II (Sotax AT7 smart, Allschwil, Switzerland) with a rotation speed of 50 rpm following the method described by Linares et al. [24]. 900 mL of distilled water at 37 ± 0.5 °C were employed as dissolution medium. About 250 mg of filament were analyzed by triplicate. 5 mL of filtered samples were withdrawn at specific interval times (15, 30, 45, 60, 120, 180, 240, 300, 360, 420, 480, 1440 min) and no fresh media was added. The percentage of AT released was measured on a UV–Vis spectrophotometer Agilent 8453 (Agilent Technologies, Santa Clara, CA, USA) at 272 nm. Taking into account the quantity of AT in the filaments (125 mg) and the existing volume during the study (considering the volume removed at each time point), less than 10% of the value of AT solubility (11.32 mg/mL) [27] was present in the medium during the whole study. So, we can confirm that the sink conditions have been maintained. 

#### 2.2.11. Dissolution Testing of 3D Printed Tablets

Dissolution studies of the 3D printed structures were carried out in the dissolution apparatus employing the same temperature and rotating speed conditions mentioned in Section 2.2.10, following the method described by Linares et al. [24]. The printed systems were first exposed to 500 mL of pH 1.2 for 2 h, simulating the stomach condition. After that, a switch solution was added to achieve pH 7.5 according to a modification of a method proposed by Schellekens et al. [28]. 5 mL of filtered samples were withdrawn at specific interval times (30, 60, 90, 120, 150, 180, 210, 240, 270, 300, 360, 420, 480, 540, 600, 660, 720 and 1440 min) and no fresh media was added. The percentage of AT released was measured on a UV–Vis spectrophotometer Agilent 8453 (Agilent Technologies, Santa Clara, CA, USA) at 272 nm. All the release data are expressed as the mean ± standard deviation (S.D.) of three samples. In the same way as in the previous section, considering the quantity of AT in the system (60 mg), sink conditions have been maintained during the study. 

AT release data (*M_t_*/*M*_∞_ ≤ 0.6) were analyzed according to zero order (2), Higuchi [29] (3), Korsmeyer et al. [30] (4) and Peppas and Sahlin [31] (5) equations:

(2) $$M_{t}/M_{\infty }=kt$$

(3) $$M_{t}/M_{\infty }=kt^{1/2}$$

(4) $$M_{t}/M_{\infty }=kt^{n}\;$$

(5) $$M_{t}/M_{\infty }=k_{d}t^{m}+k_{r}t^{2m}$$

where $M_{t}/M_{\infty }$ is the AT released fraction at time *t* (*M_∞_* corresponds to the 100% of AT released), *k* is the zero-order release constant on Equation (2), the Higuchi release rate constant on Equation (3) and the Korsmeyer kinetic constant on Equation (4), respectively. *t* is the release time, *n* is the release exponent that depends on the release mechanism and the shape of the matrix tested [32], *k_d_* is the diffusional constant and *k*_r_ the relaxational rate constant. *m* is the purely Fickian diffusion exponent (which depends on the geometrical shape of the releasing device through its aspect ratio).

The optimum values for the parameters present in each equation were determined using Microsoft Excel 2010 (Microsoft, Albuquerque, NM, USA). The determination coefficient (r^2^) was used to test the applicability of the release models, calculated in linear regression for zero order, Higuchi and Korsmeyer equations or quadratic regression for Peppas and Sahlin model.

## 3. Results

Regarding the extrusion process, binary mixtures were extruded at 130 °C. The extrusion temperature decreased as the AT concentration increased for ternary mixtures, from 150 °C to 135 °C. A single screw extruder was employed for all the batches. Binary and ternary filaments showed suitable diameter to be printed by FDM (1.69–1.82 mm for binary filaments and 1.70–1.78 mm for ternary filaments), with the exception of B30 and B40 batches that had a slightly smaller diameter than the lower limit (1.56–1.64 mm). Filament diameters from 1.75 ± 0.1 mm can be accepted since they allow the drive wheels to print with accuracy [24].

A stiffness test was carried out to measure the mechanical behaviour of the filaments. This test allows to measure the property toughness, which has been related to the ability of a filament to be printed by means of a FDM 3D printer. 

**Figure 2:**
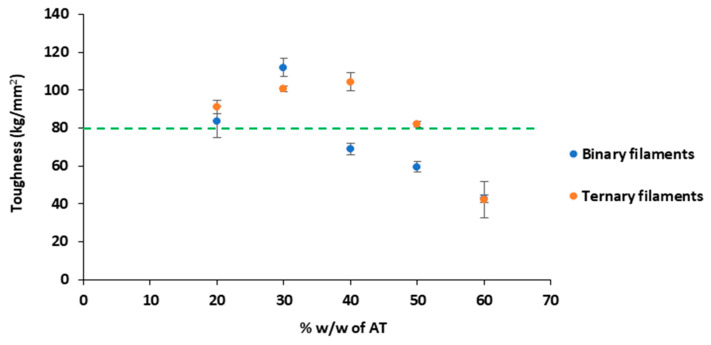
Graphical representation of the toughness versus the percentage *w*/*w* of AT in the filaments prepared.

According to Xu et al. [26] a FDM printability threshold is established above a toughness value of 80 kg/mm^2^ for the filaments. As shown in Figure 2, binary filaments below 30% *w*/*w* of AT and ternary filaments below 50% *w*/*w* of AT have a toughness value above this critical point due to the higher content in thermoplastic polymer. In the case of ternary filaments, the effect of magnesium stearate improves the toughness value for filaments containing up to 50% *w*/*w* of AT. These results fit with the performance observed during the 3D printing process. Filaments with low values of toughness had poor mechanical properties and failed to be printed.

Morphological analysis of the extruded filaments was carried out based on the fractal analysis in order to measure its roughness. Values of the linear fractal dimension nearer to 1 are indicative of lower roughness [33].

**Figure 3:**
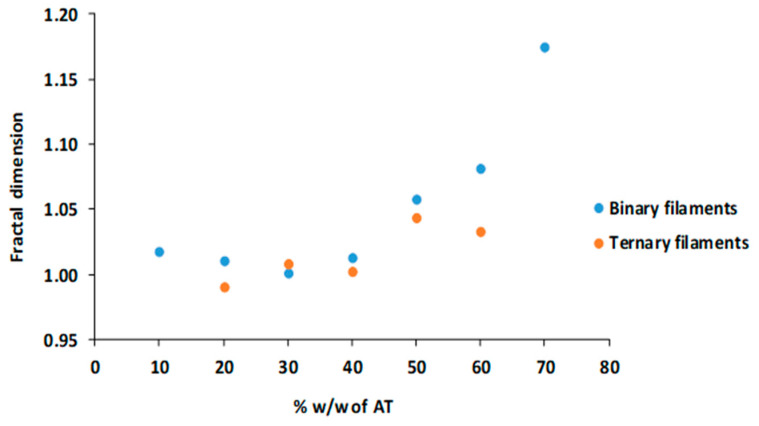
Graphical representation of the fractal dimension versus the percentage *w*/*w* of AT in the filaments prepared.

It can be seen in Figure 3 that the ternary batches containing magnesium stearate show lower values of the fractal dimension compared to the binary filaments. These data confirm the lower roughness of the ternary filaments since the presence of magnesium stearate softens the surface of the filament.

Figure 3 also illustrates that binary filaments showed a decreasing roughness between 20–30% *w*/*w* of AT content and then increased for the 40–60% *w*/*w*. This change in roughness was related to the ability of these binary filaments to be 3D printed using the FDM technique, since only filaments up to 30% *w*/*w* of AT could be used for the printing process. These results are in agreement with those obtained by Mora-Castaño et al. [15]. In the case of ternary filaments, it cannot be appreciated a clear relationship between the roughness and the percentage of AT since the use of magnesium stearate as lubricant masks this effect making the surface of the filaments soften. In general, the roughness decreases even though they have a high percentage of AT when compared to binary filaments, and it is also possible to use filaments up to 50% *w*/*w* of AT for 3D printing.

Based on the results of the fractal analysis and the mechanical properties of the filaments, batch T50 consisting of a ternary blend of AT, thermoplastic polyurethane and magnesium stearate (50-45-5% *w*/*w*) was selected as the filaments containing the highest amount of AT able to be printed by FDM 3D printing.

Results from the Differential Scanning Calorimetry and Thermogravimetric Analysis of the pure components, physical mixtures containing 50-45-5% *w*/*w* of AT, thermoplastic polyurethane and magnesium stearate, T50 filaments and 3D printed systems confirm the stability and compatibility of the materials used (see Supplementary Data).

**Figure 4:**
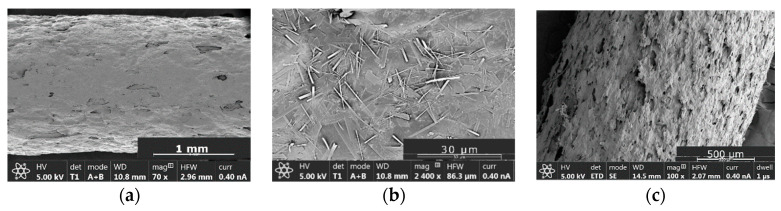
Microphotographs of filaments. (**a**) Microphotograph of the surface of a T50 filament; (**b**) Detail of the surface of a T50 filament where AT particles can be appreciated; (**c**) Microphotograph of the surface of a B50 filament.

The morphology of the filaments containing 50% *w*/*w* of AT was analyzed by SEM (see Figure 4). It can be observed that a suspension-type solid dispersion with AT particles distributed in the continuous medium formed by TPU has been obtained (see Figure 4b). It is expected that the continuum percolation model, where the percolation threshold of a substance is around 16% *v*/*v* of its occupation probability or volume concentration, could explain the behaviour of the filaments. Therefore, 45% *w*/*w* of TPU is expected to form a coherent matrix which controls the AT release. It can be observed that the roughness of the T50 filaments is quite lower compared with the B50 filaments, containing both 50% of AT, being the only difference the absence of magnesium stearate in batch B50 (Figure 4a,c). 

**Figure 5:**
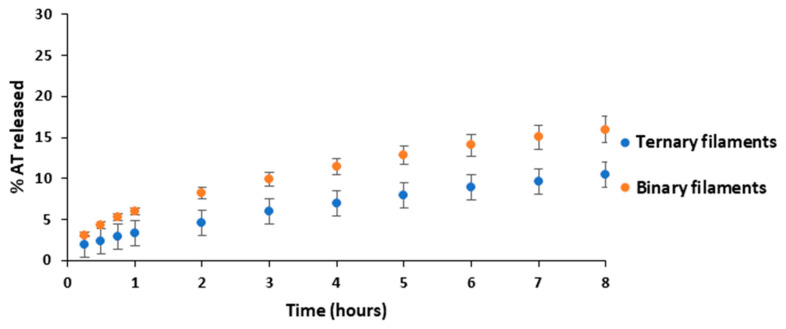
AT release profiles from B50 filaments and T50 filaments.

Figure 5 depicts the AT released from B50 and T50 filaments. The AT released from the T50 filaments was slower in comparison to the B50 filaments that contained the same amount of AT. T50 filaments released around 10% of AT after 8 h of assay while B50 filaments released approximately 16% of AT in the same period. It is reasonable since the hydrophobic character of magnesium stearate slowdown the AT release. The low percentage of AT released in both cases is due to the fact that the filaments have a low surface exposed to the dissolution medium, making difficult the release of the AT that is not in contact with the filament surface.

T50 filaments were successfully printed in a Raise^®^ FDM 3D printer, resulting in a structure containing 61.21 ± 0.07 mg of AT. This way, a higher AT load is obtained in comparison with the structures in which the AT is injected by IVF [19].

**Figure 6:**
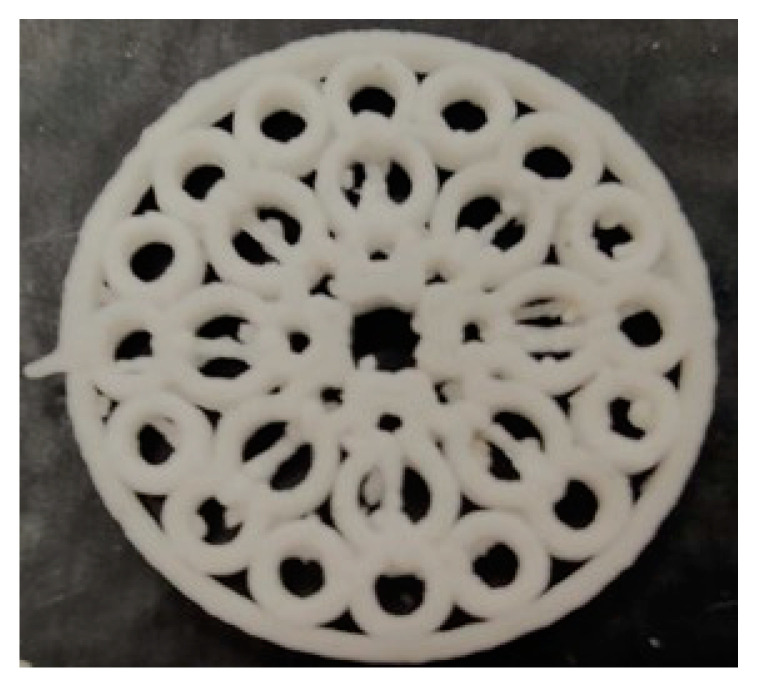
Photograph of the 3D printed structure.

In order to maximize the surface in contact with the dissolution medium and the subsequent water uptake, a large number of pores were included in the flat structure. An image of the fractal-like structure can be appreciated in Figure 6. The weight and dimensions of the systems according to the digital design are recorded in Table 2.

As it can be appreciated, a high uniformity of weight and dimensions is obtained for the 3D printed structures. This fact is indicative of the high reproducibility of the 3D printing process. Moreover, an excellent concordance between the diameters designed and obtained was found in the printing process, showing a high accuracy and precision of the manufacturing FDM technology. However, in the case of the height, it can be observed a slight difference between the theoretical and the experimental dimensions. It can be due to the fact that the parameters employed for the 3D printing process promote an increment of the flow through the nozzle to improve the adhesion of the structure to the building platform.

Moreover, the printing process takes place at a relatively high speed (the building of the FDM structure takes only six minutes).

**Figure 7:**
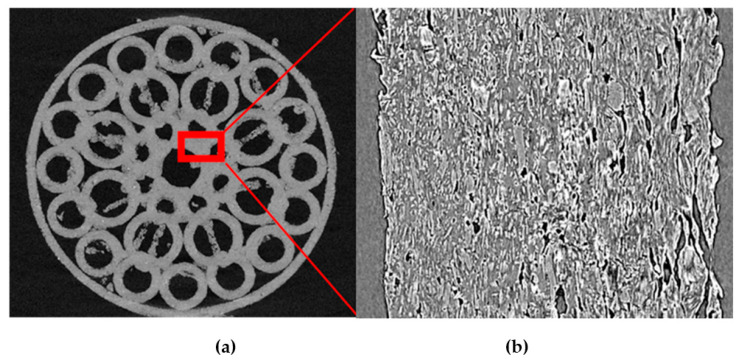
X-ray tomography. (**a**) Image of the 3D printed structure. (**b**) Detailed of one toroid of the structure.

The Figure 7 illustrates the X-ray tomography of the FDM printed structure and a detail of one of the toroids where a homogeneous distribution of the AT in the polymer matrix can be observed. The non-molten AT can be easily appreciated as the particles with acicular shape. On the other hand, the molten polymer corresponds to the darkest background. 

**Figure 8:**
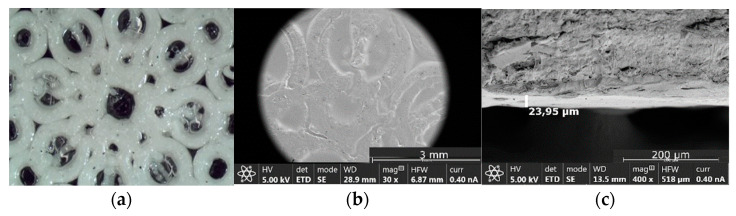
Microphotographs of the 3D printed structures. (**a**) Fractal-like coated structure (optical microscopy); (**b**) SEM microphotograph of the surface of the 3D printed coated structure; (**c**) SEM microphotograph of the cross section of a coated structured.

A coating process with Drugcoat S 12.5 was carried out using IVF technology. The coating polymer is a methacrylic acid–methyl methacrylate Copolymer (1:2) in 12.5% organic solution which dissolves at pH values above 7. The original design of the structure allowed the complete coating using a simple top injection with the liquid polymer which completely covered the system through the pores. The formation of a homogeneous Drugcoat transparent film can be observed in Figure 8a. Figure 8b shows a SEM microphotograph of the coated structure where the integrity of the enteric coating can also be appreciated. Figure 8c illustrates a microphotograph of the cross section of a coated structure, showing the thickness of the coating layer (23.95 µm). The coating process by IVF takes 3 min.

**Figure 9:**
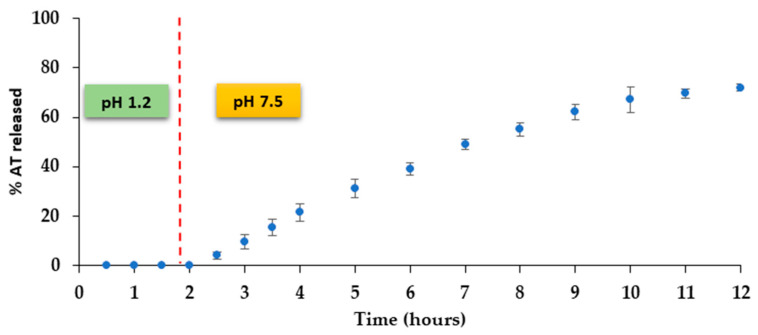
AT release profile from the 3D printed system.

The percentage of AT released versus time from the printed systems is showed in Figure 9. It can be noticed that there is an absence of AT release during the 2 h that the structure is at pH 1.2, simulating the gastric residence time. The AT release automatically starts once the pH of the dissolution medium increases to 7.5. This fact reveals that the integrity of the enteric coating is perfectly maintained during the time that the dosage form is in contact with the acidic medium. The enteric film dissolves immediately after the system is in contact with the basic medium. 

A prolonged cumulative release profile can be observed for the 3D printed systems that release around 70% of AT after 12 h. Additionally, a constant AT release is achieved from 2 to 7 h. There is a constant search for drug delivery systems that release with zero order kinetics due to the advantages associated to these dosage forms [34]. With the emergence of 3D printing technologies, different approaches have been tested in order to achieve a constant drug release. Some examples are based on a non-uniform distribution of the drug in the system or a control of the swelling behavior [35]. In our case, a modulation of the geometry of the release area, based on multiple toroids has been carried out in order to expose the maximum surface to the release medium. As a consequence, the diffusion pathway of the drug in the structure is quite short and uniform. Moreover, the burst release observed in other similar geometric structures -such as intravaginal rings [36,37] is avoided, maybe due to the high ability of the TPU to control the AT release.

Release data analysis was carried out according to zero order, Higuchi [29], Korsmeyer et al. [30] and Peppas and Sahlin [31] equations with the aim of understanding the AT release process from the printed structures. As can be observed in Table 3, the system prepared shows a value of Korsmeyer’s time exponent *n* very close to 1, which can be attributed to zero-order release kinetics. A good adjustment to the zero-order release kinetics can also be assumed from the value of the determination coefficient (0.9947). According to the Peppas and Sahlin equation, a higher value for the diffusional constant can be observed, indicating the predominance of the diffusion mechanism for the AT release.

## 4. Conclusions

A 3D printed system with high AT load intended for zero-order colonic release has been obtained using the combination of FDM and IVF technologies. The mechanical characterization of the filaments provided a useful information for the printing process. The incorporation of 5% *w*/*w* of magnesium stearate in the TPU filaments allowed loading 60 mg of AT. This result, which represents the 50% of the mass of the FDM structure, is much higher than those obtained by IVF in previous works.

Regarding the original design of the 3D printed structure, it permits the homogeneous coating injecting the enteric polymer in a unique step. This method avoids a subsequent coating process in a fluid bed coater which would imply the preparation of a batch of higher size and a longer time. Moreover, a prolonged and constant AT release was obtained once the enteric polymer was dissolved. 

The combination of FDM and IVF has demonstrated to be suitable for the manufacture of colonic high drug loaded delivery systems in a short period of time. These systems can be very useful to be implemented in hospitals for on-demand dispensing of personalized medicines.

## Figures and Tables

**Table 1:** Composition of the prepared batches.

Batch	Theophylline Anhydrous (AT) (%)	Thermoplastic Polyurethane (TPU) (%)	Magnesium Stearate (%)
B20	20	80	0
B30	30	70	0
B40	40	60	0
B50	50	50	0
B60	60	40	0
T20	20	75	5
T30	30	65	5
T40	40	55	5
T50	50	45	5
T60	60	35	5

**Table 2:** Physical characteristics of the 3D printed structure.

		Experimental (*n* = 6)		Theoretical	
	Height (mm)	Diameter (mm)	Weight (mg)	Height (mm)	Diameter (mm)
Mean	1.22	15.02	120.32	1	15
SD	0.04	0.05	4.26		

**Table 3:** AT release kinetics from the 3D printed structures.

Higuchi		Korsmeyer			Zero Order		Peppas and Sahlin		
b ^a^ (min^−0.5^)	r^2 e^	K ^a^ (min^−n^)	n ^b^	r^2 e^	K ^a^ (min^−1^)	r^2 e^	k_d_ ^c^ (min^−0.425^)	k_r_ ^d^ (min^−0.850^)	r^2 e^
0.0331	0.9433	0.00147	1.0166	0.9917	0.0016	0.9947	0.0065	0.0012	0.996

^a^ Higuchi, Korsmeyer and Zero order kinetic constant; ^b^ release exponent; ^c^ Peppas diffusion kinetic constant. ^d^ Peppas relaxation kinetic constant; ^e^ determination coefficient.

## Data Availability

Not applicable.

## Supplementary Materials

The following are available online at https://www.mdpi.com/article/10.3390/pharmaceutics14112298/s1, DSC thermograms an TGA thermograms.
