# Supplementary material for: 3D Printed Fractal-like Structures with High Percentage of Drug for Zero-Order Colonic Release
Source: Pharmaceutics. 2022 Oct 26;14(11):2298. doi: 10.3390/pharmaceutics14112298 (PMC9695807; doi:10.3390/pharmaceutics14112298)
Supplement: Supplementary file 1 [file pharmaceutics-14-02298-s001.zip › Figure 10. TGA of the physical mixture.pdf]

Sample: Mezcla
Size: 10.4720 mg

DSC-TGA

File: E:...\SCF-4153-22\Mezcla.001
Operator: Lola
Run Date: 20-Jul-2022 10:58
Instrument: SDT Q600 V20.9 Build 20

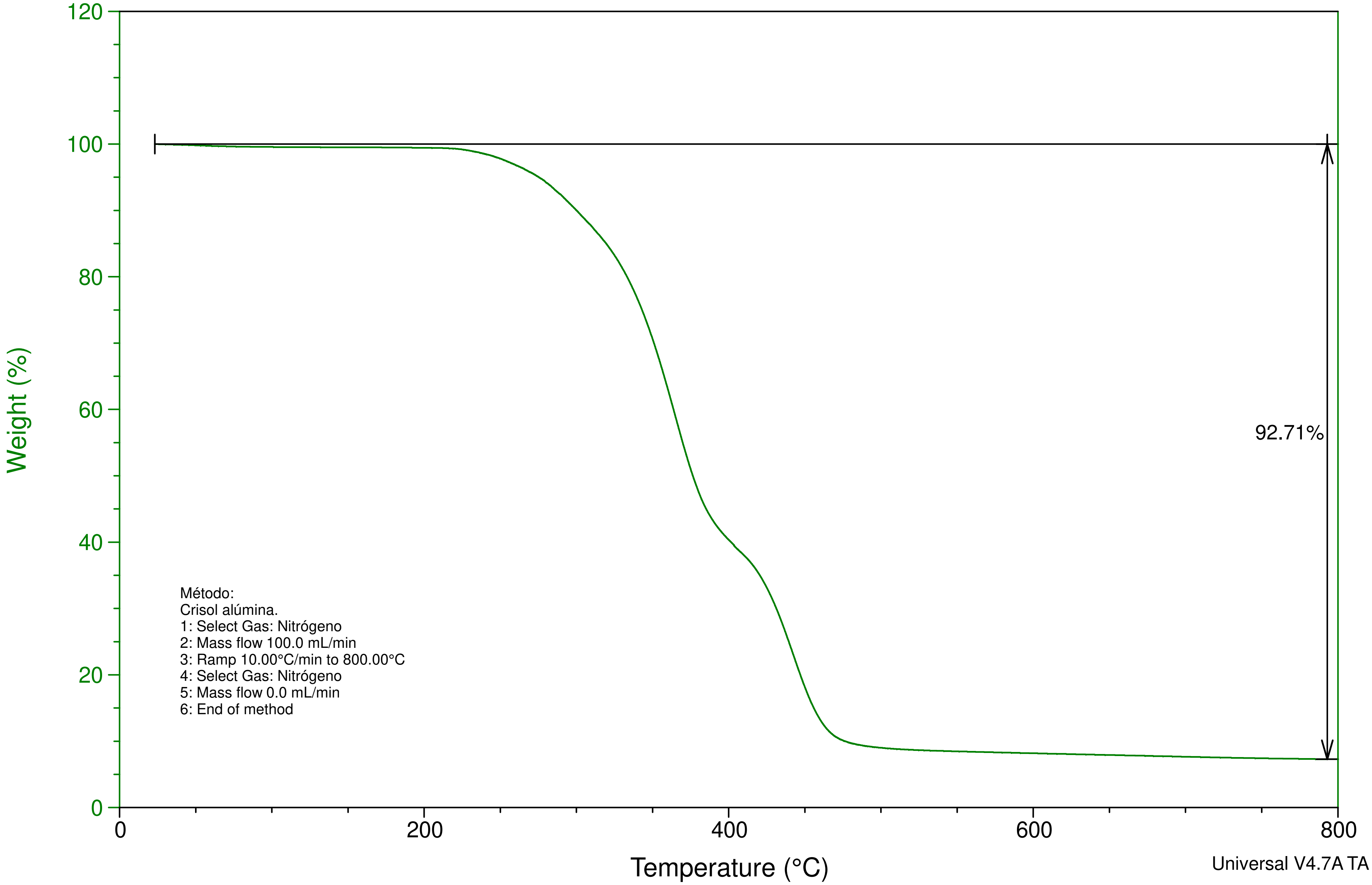

Universal V4.7A TA Instruments
