# Supplementary material for: 3D Printed Fractal-like Structures with High Percentage of Drug for Zero-Order Colonic Release
Source: Pharmaceutics. 2022 Oct 26;14(11):2298. doi: 10.3390/pharmaceutics14112298 (PMC9695807; doi:10.3390/pharmaceutics14112298)

Sample: Teofilina
Size: 3.8000 mg

DSC

File: E:...\SCF-4154-22\Teofilina.001
Operator: Lola
Run Date: 21-Jul-2022 14:18
Instrument: DSC Q20 V24.11 Build 124

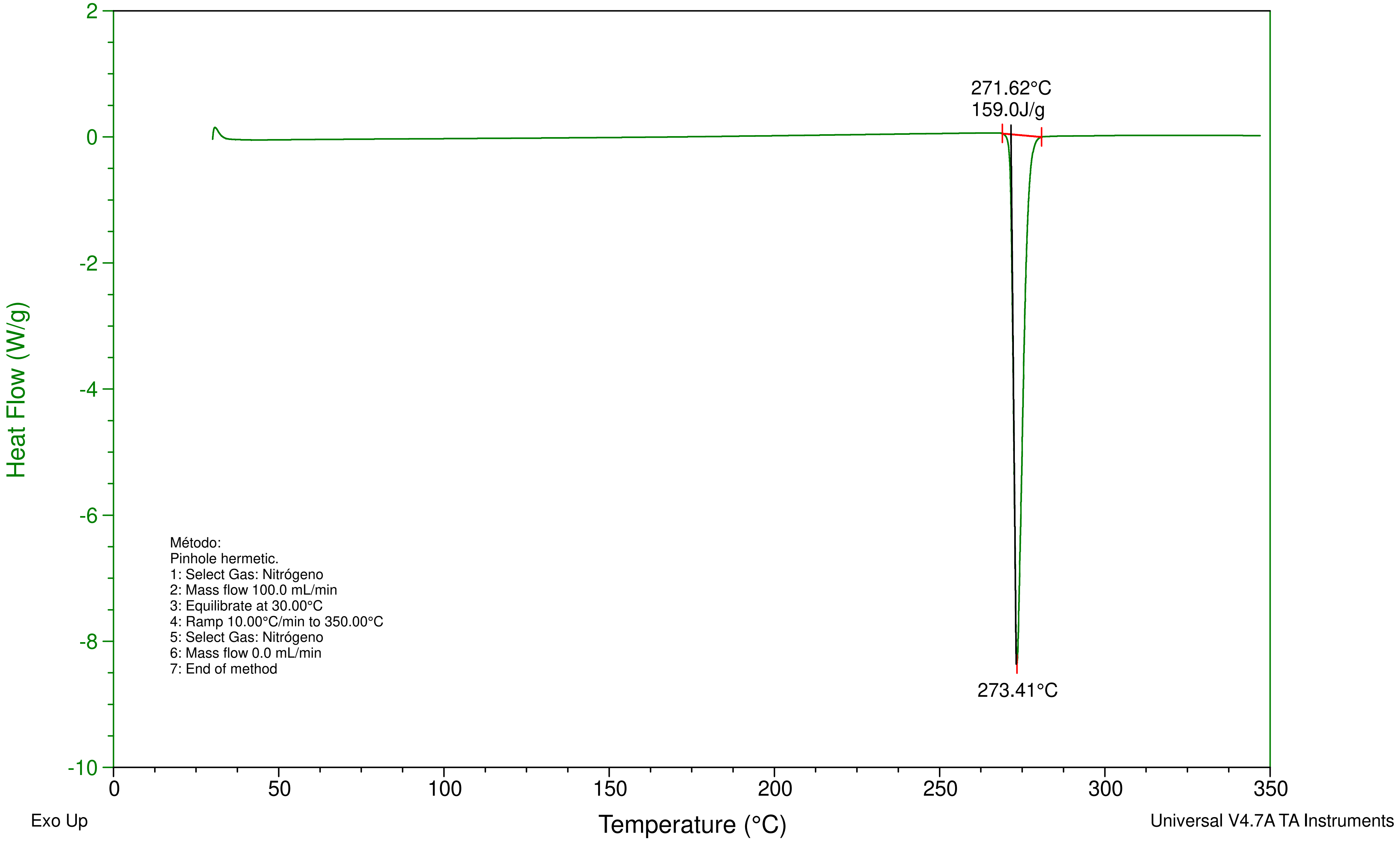

Supplement: Supplementary file 1 [file pharmaceutics-14-02298-s001.zip › Figure 3. DSC of Theophylline.pdf]
