# Supplementary material for: 3D Printed Fractal-like Structures with High Percentage of Drug for Zero-Order Colonic Release
Source: Pharmaceutics. 2022 Oct 26;14(11):2298. doi: 10.3390/pharmaceutics14112298 (PMC9695807; doi:10.3390/pharmaceutics14112298)
Supplement: Supplementary file 1 [file pharmaceutics-14-02298-s001.zip › Figure 9. TGA of Theophylline.pdf]

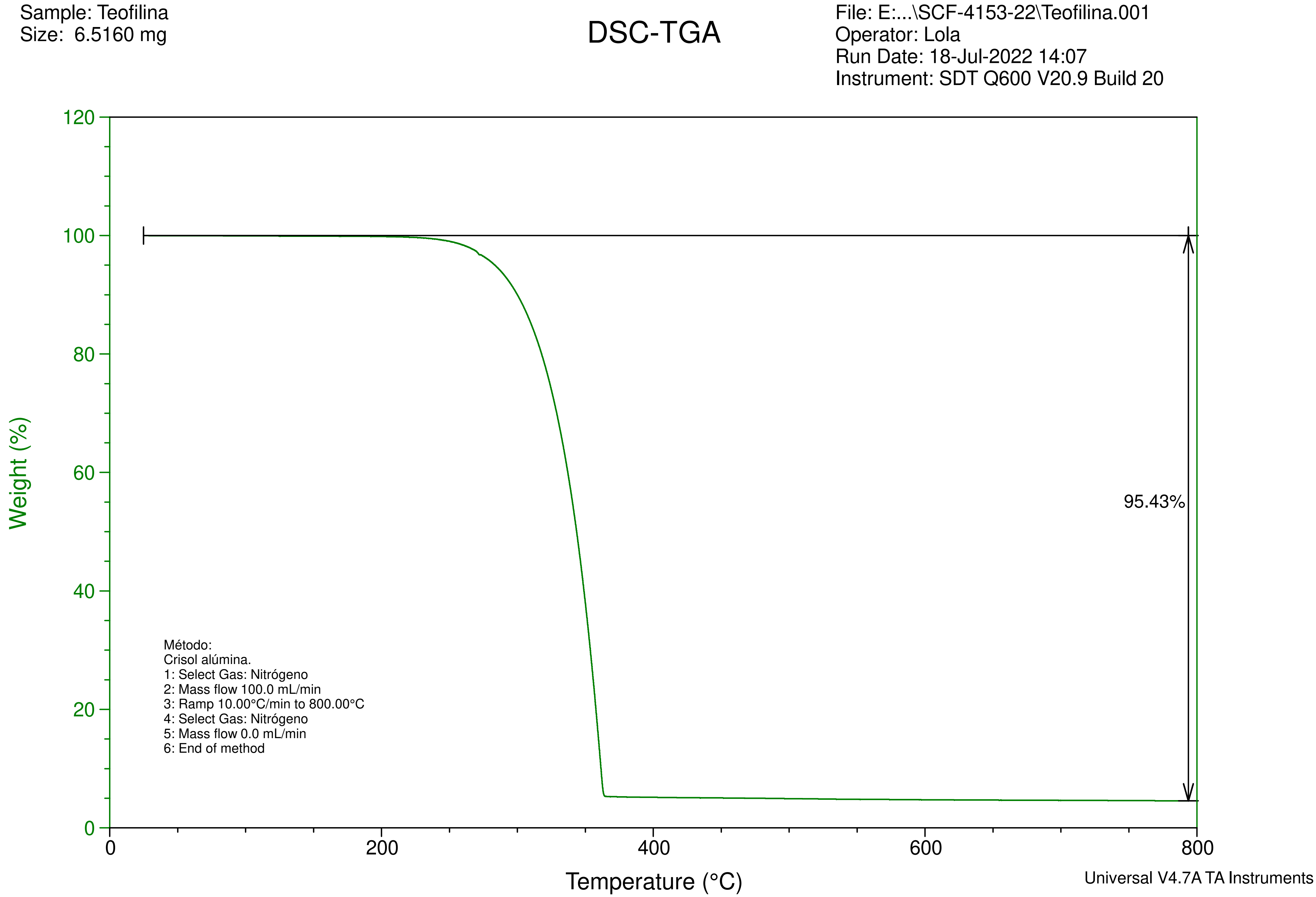
Sample: Teofilina
Size: 6.5160 mg
DSC-TGA
File: E:...\SCF-4153-22\Teofilina.001
Operator: Lola
Run Date: 18-Jul-2022 14:07
Instrument: SDT Q600 V20.9 Build 20
Weight (%)
120
100
80
60
40
20
0
95.43%
Método:
Crisol alúmina.
1: Select Gas: Nitrógeno
2: Mass flow 100.0 mL/min
3: Ramp 10.00°C/min to 800.00°C
4: Select Gas: Nitrógeno
5: Mass flow 0.0 mL/min
6: End of method
0
200
400
600
800
Temperature (°C)
Universal V4.7A TA Instruments
