# Supplementary figures and images for: 3D Printed Fractal-like Structures with High Percentage of Drug for Zero-Order Colonic Release
Source: Pharmaceutics. 2022 Oct 26;14(11):2298. doi: 10.3390/pharmaceutics14112298 (PMC9695807; doi:10.3390/pharmaceutics14112298)

Sample: EG72  
Size: 3.5000 mg

## DSC

File: E:\...\SCF-4154-22\EG72.001  
Operator: Lola  
Run Date: 22-Jul-2022 11:23  
Instrument: DSC Q20 V24.11 Build 124

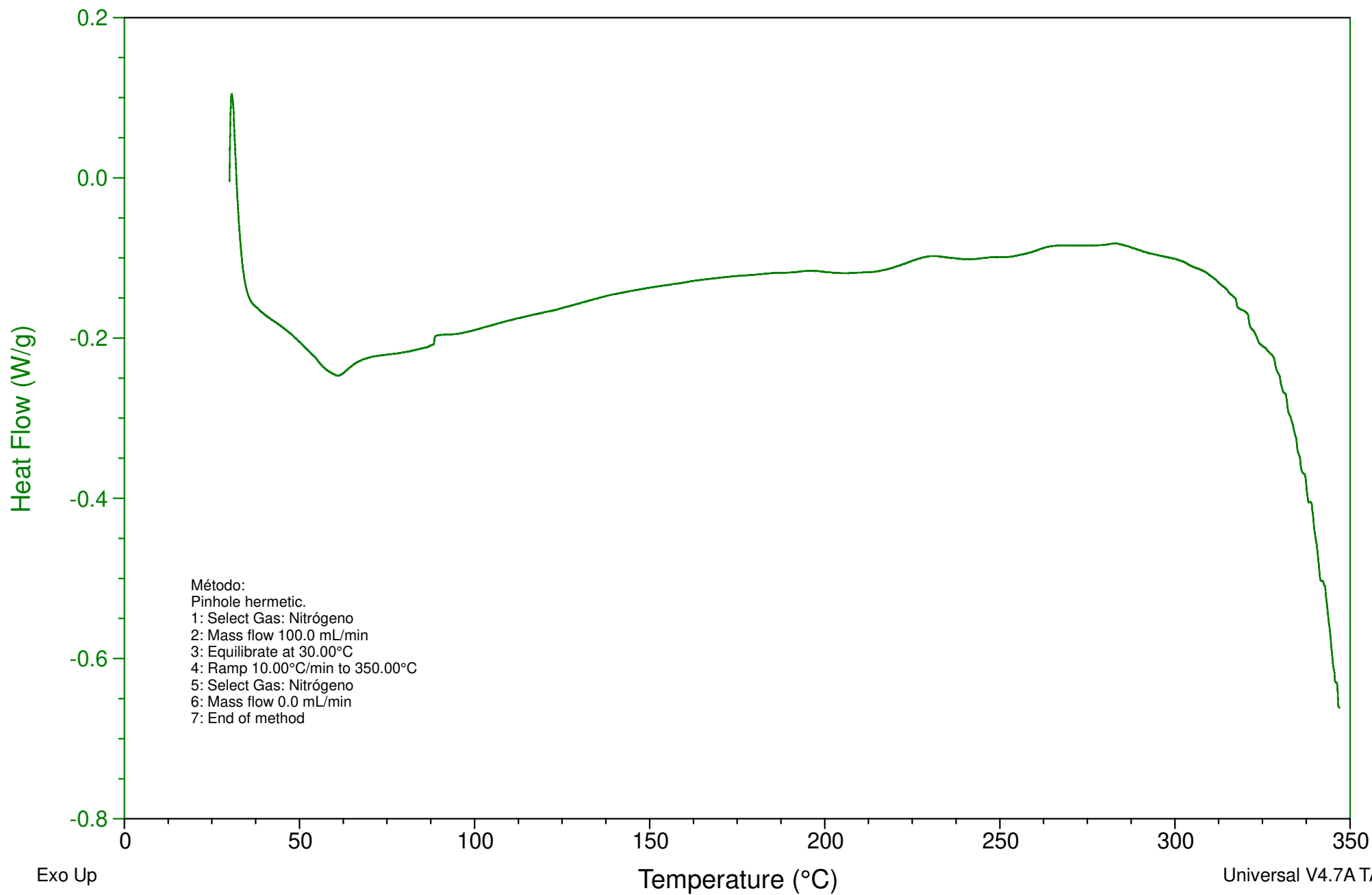

Supplement: Supplementary file 1 [file pharmaceutics-14-02298-s001.zip › Figure 1. DSC of TPU Tecoflex EG72D.pdf]

Sample: Mg St  
Size: 3.3000 mg

# DSC

File: E:\...\SCF-4154-22\Mg St.001  
Operator: Lola  
Run Date: 21-Jul-2022 16:18  
Instrument: DSC Q20 V24.11 Build 124

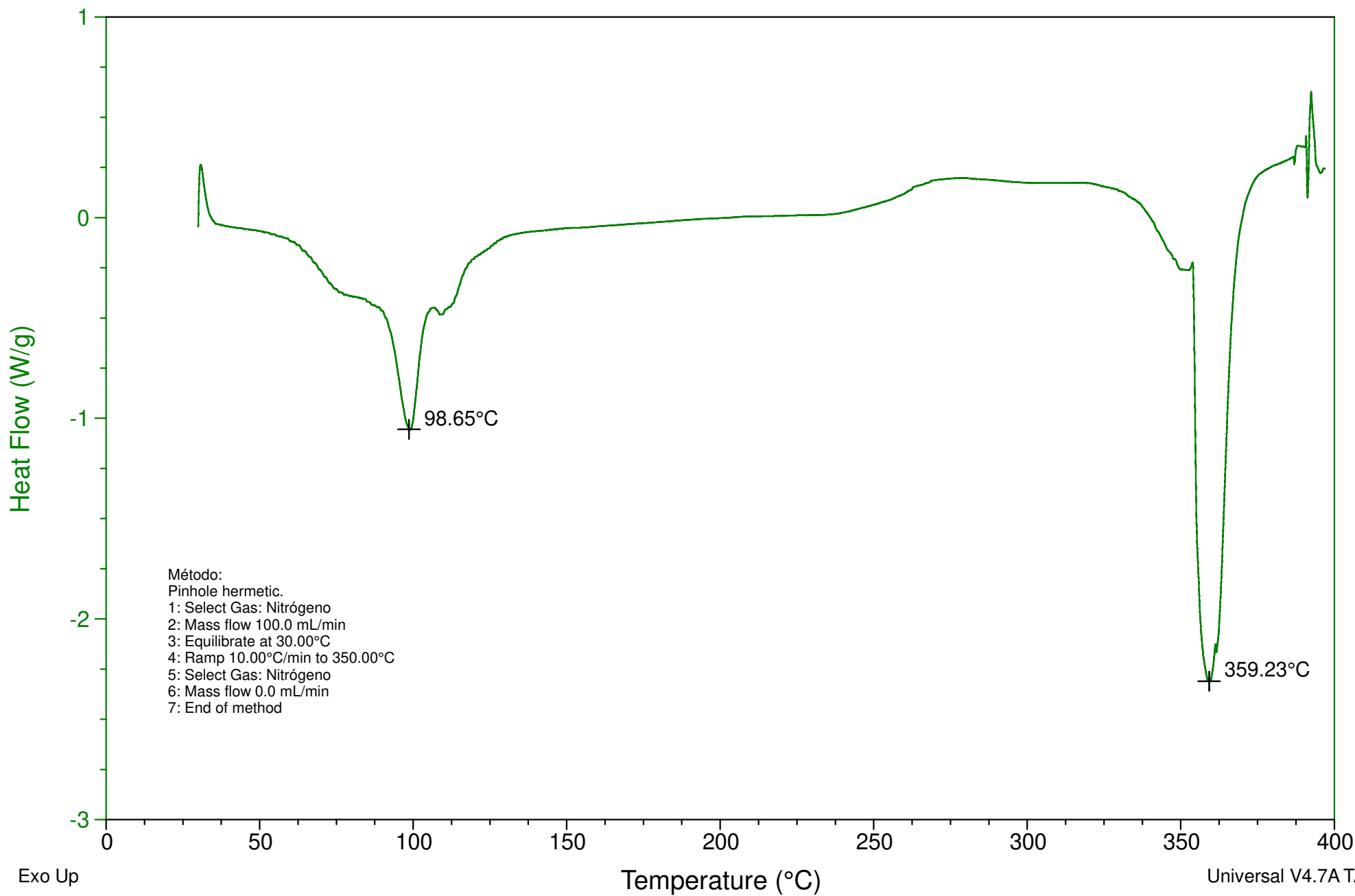

Supplement: Supplementary file 1 [file pharmaceutics-14-02298-s001.zip › Figure 2. DSC of Magnesium stearate.pdf]

Sample: Mezcla  
Size: 3.8000 mg

# DSC

File: E:\...\SCF-4154-22\Mezcla.001  
Operator: Lola  
Run Date: 21-Jul-2022 14:58  
Instrument: DSC Q20 V24.11 Build 124

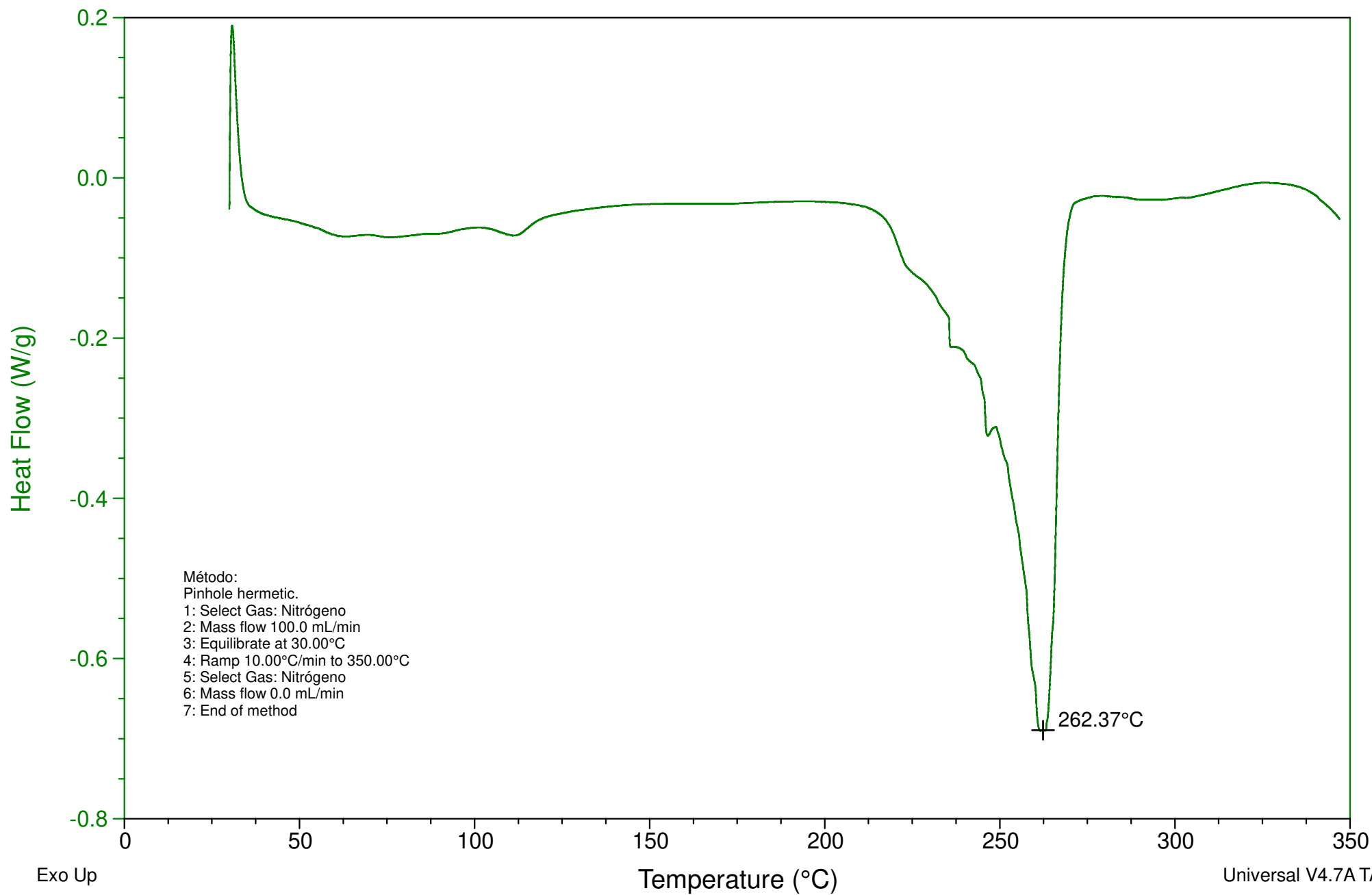

Supplement: Supplementary file 1 [file pharmaceutics-14-02298-s001.zip › Figure 4. DSC of the physical mixture.pdf]

Sample: Mandala  
Size: 2.9000 mg

# DSC

File: E:\...\SCF-4154-22\Mandala.001  
Operator: Lola  
Run Date: 21-Jul-2022 12:57  
Instrument: DSC Q20 V24.11 Build 124

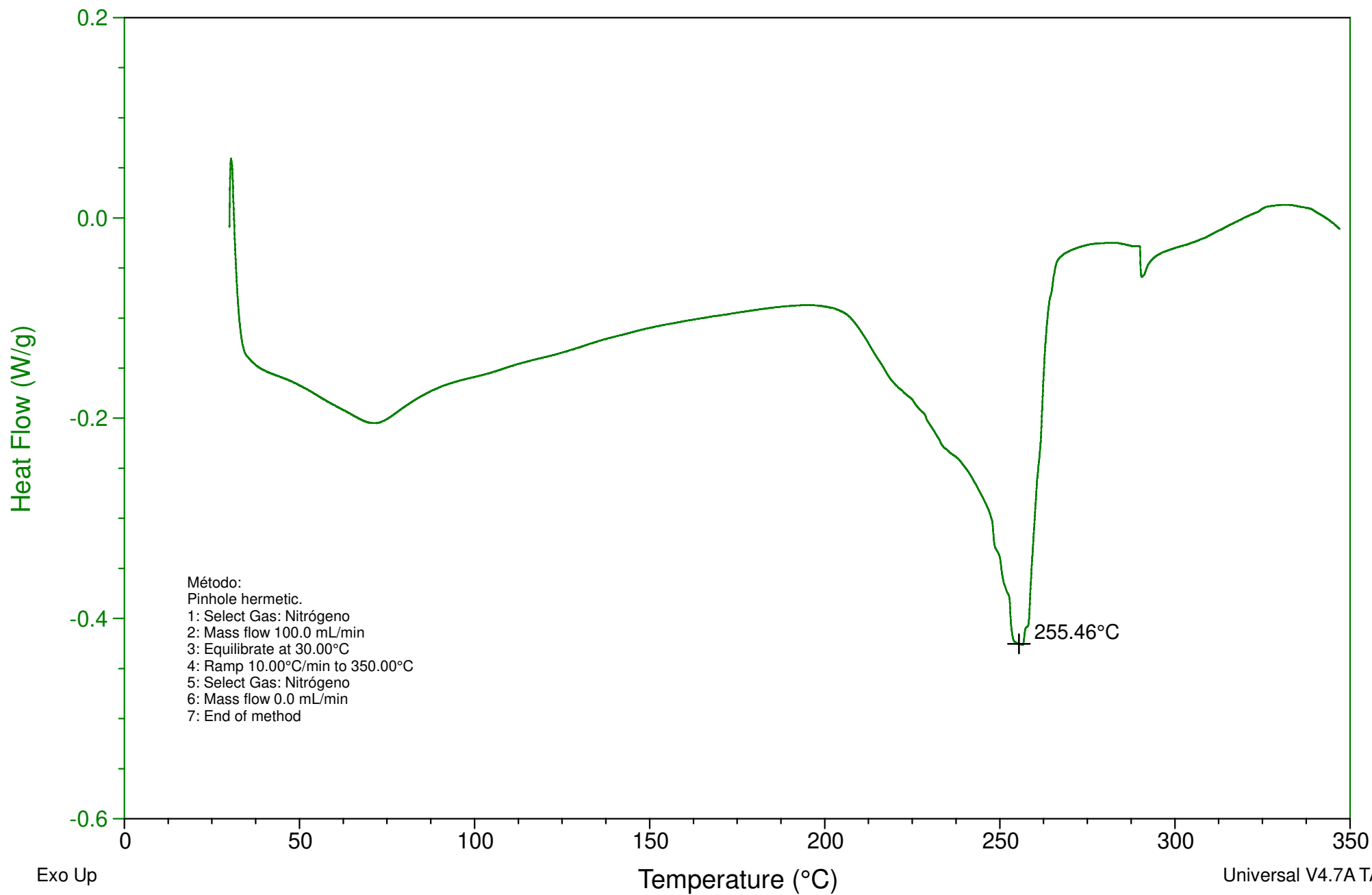

Supplement: Supplementary file 1 [file pharmaceutics-14-02298-s001.zip › Figure 6. DSC of the 3D printed structure.pdf]

Sample: EG72  
Size: 6.3040 mg

DSC-TGA

File: E:\...\SCF-4153-22\EG72.001  
Operator: Lola  
Run Date: 18-Jul-2022 10:28  
Instrument: SDT Q600 V20.9 Build 20

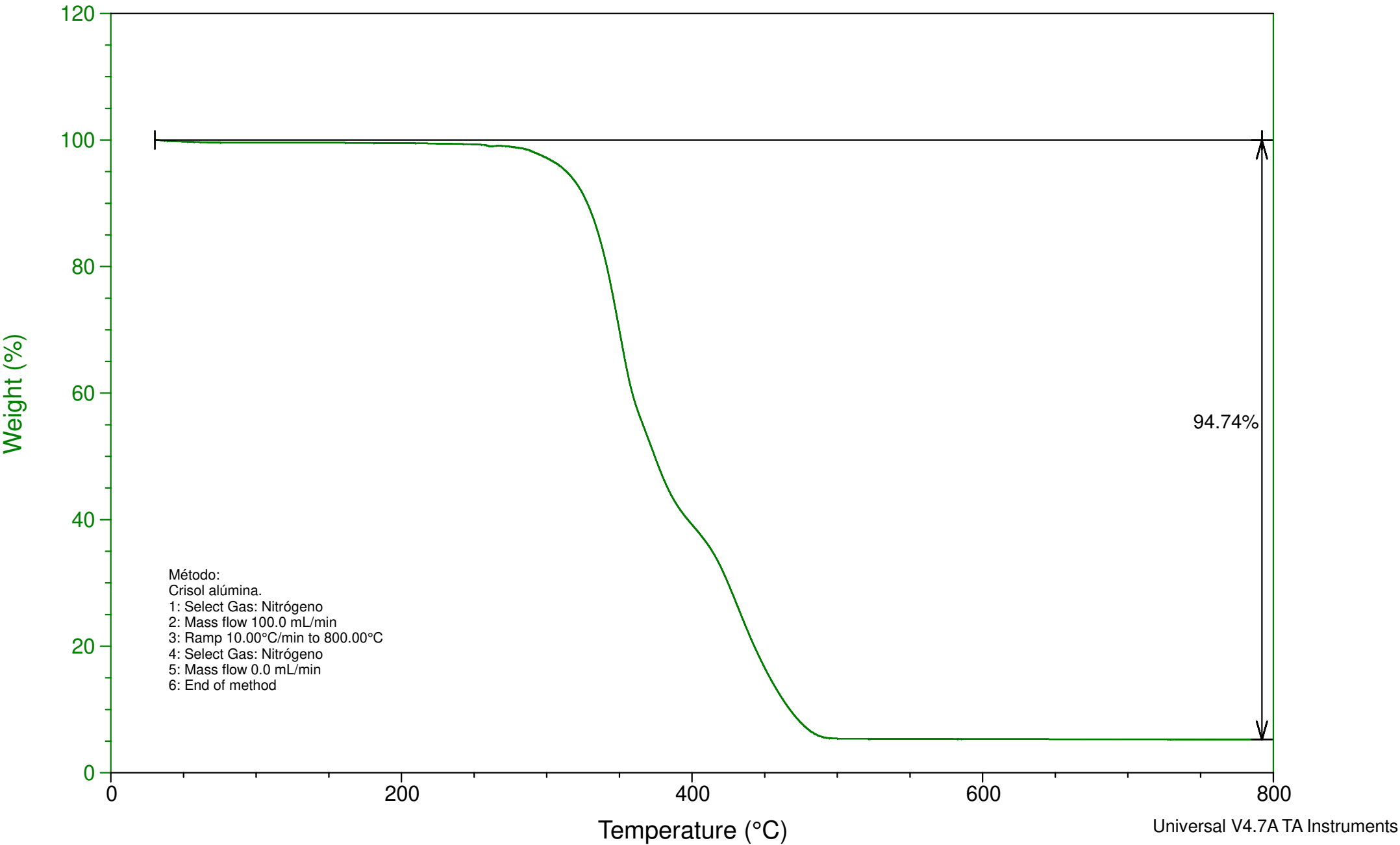

Supplement: Supplementary file 1 [file pharmaceutics-14-02298-s001.zip › Figure 7. TGA of TPU Tecoflex EG72D.pdf]

Sample: Mg St  
Size: 4.3530 mg

DSC-TGA

File: E:\...\SCF-4153-22\Mg St.001  
Operator: Lola  
Run Date: 19-Jul-2022 08:14  
Instrument: SDT Q600 V20.9 Build 20

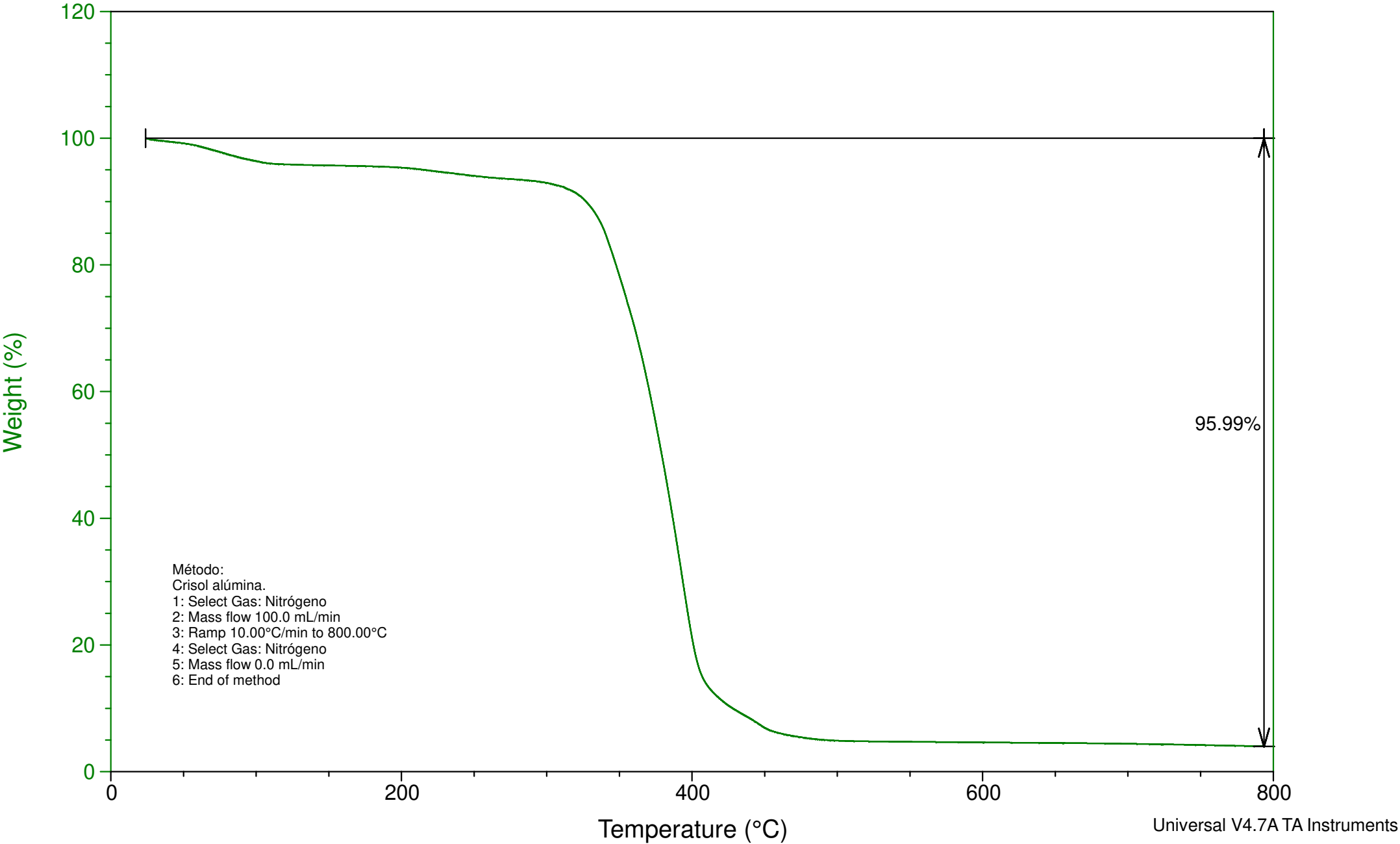

Supplement: Supplementary file 1 [file pharmaceutics-14-02298-s001.zip › Figure 8. TGA of Magnesium stearate.pdf]
